# Icteric Variant of Stauffer Syndrome as a Paraneoplastic Manifestation of Type 1 Papillary Renal Cell Carcinoma

**DOI:** 10.14309/crj.0000000000001111

**Published:** 2023-08-02

**Authors:** Juan Antonio Sorda, Fernando Javier Barreyro, German Rojas, Daniel Alejandro Greco, Andrea Paes, Alejandra Avagnina, Jorge Daruich, Esteban González Ballerga

**Affiliations:** 1 Department of Gastroenterology and Hepatology, University Hospital “José de San Martín”, Faculty of Medicine, University of Buenos Aires, Argentina; 2 Laboratory of Molecular Biotechnology (BIOTECMOL), Biotechnology Institute of Misiones (INBIOMIS), National University of Misiones, National Scientific and Technical Research Council (CONICET), Argentina; 3 Department of Pathology, University Hospital “José de San Martín,” Faculty of Medicine, University of Buenos Aires, Argentina

**Keywords:** cholestasis, paraneoplastic syndrome, papillary renal cell carcinoma

## Abstract

Intrahepatic cholestasis as a paraneoplastic manifestation was first described by Dr. Maurice H. Stauffer in 1961. This paraneoplastic manifestation was primarily associated with renal cell carcinoma characterized by abnormal liver enzymes without hepatic metastasis. Stauffer syndrome is classified into 2 types: classical and jaundice variants. Indeed, the jaundice variant is extremely rare and only described in 13 published cases. We report a case of intrahepatic cholestasis associated with a type 1 papillary renal cell carcinoma with complete resolution after surgical treatment.

## INTRODUCTION

Neoplasm-related cholestasis mainly occurs from widespread liver metastases or because of tumoral compression/infiltration of the bile ducts.^1^ Intrahepatic cholestasis as a paraneoplastic manifestation is rarely seen.^1^ Paraneoplastic syndromes constitute a heterogeneous group of signs and symptoms associated with a malignant neoplasm, and they probably arise from tumor secretion of hormones, peptides, and cytokines or from immune-mediated injury.^2^ The syndrome may occur at any stage of primary tumor, affects patient's quality of life, and in some cases, could affect survival.^2^

Intrahepatic cholestasis as a paraneoplastic manifestation associated with renal cell carcinoma was first described by Dr. Stauffer in 1961 and later on was observed in other neoplasms.^3–6^ The anicteric cholestasis variant is the most common form of presentation while icteric cholestasis is extremely rare.^7,8^ We present a case of Stauffer syndrome (SS) associated with a type 1 papillary renal cell carcinoma (pRCC) with clinical presentation of jaundice and resolution of cholestasis after surgical treatment.

## CASE REPORT

A 67-year-old man was referred to our hospital with insidious itching, scleral icterus, progressive dark urine, and hypocholia. Physical examination revealed jaundice, hepatomegaly, and mild lower limb edema. He had no known liver disease but had hypertension, type II diabetes mellitus, and obesity. He had been taking carvedilol, enalapril, and metformin. There were no other new medications or alcohol or illicit drug use.

Laboratory studies were remarkable for abnormal liver function tests with a cholestatic pattern and elevation of serum bile acids (total bilirubin: 11.5 mg/dL, conjugated bilirubin: 11.2 mg/dL, aspartate aminotransferase: 328 UI/mL, alanine aminotransferase: 73 UI/mL, alkaline phosphatase: 1,559 UI/mL, gamma glutamyl transferase: 894 UI/mL, serum bile acid [BA] 37.5 μmol/L). Serological workup for infectious agents (hepatitis B surface antigen, anti-IgM antibody against hepatitis B core antigen, anti-IgM antibody hepatitis A virus, anti-IgM antibody against hepatitis E virus, anti-antibodies against hepatitis C virus and anti-HIV) and autoimmune etiologies (anti-smooth muscle antibody, anti-liver-kidney microsomal antibody, anti-mitochondrial antibody, anti-nuclear antibody, anti-neutrophil cytoplasmic antibodies and immunoglobulin G subtype-4) were negative. Abdominal ultrasound and magnetic resonance cholangiopancreatography revealed hepatomegaly, without focal liver lesions and no evidence of bile duct injury or choledocholithiasis. An incidental right upper pole renal mass was observed, isointense in T1 and hyperintense in T2 of 48 and 33 mm in diameter, respectively, that resembled primary renal tumor. Tumor staging was performed by computed tomography scan of the thorax, abdomen, and pelvis and showed no evidence of metastatic spread.

**Figure 1.**
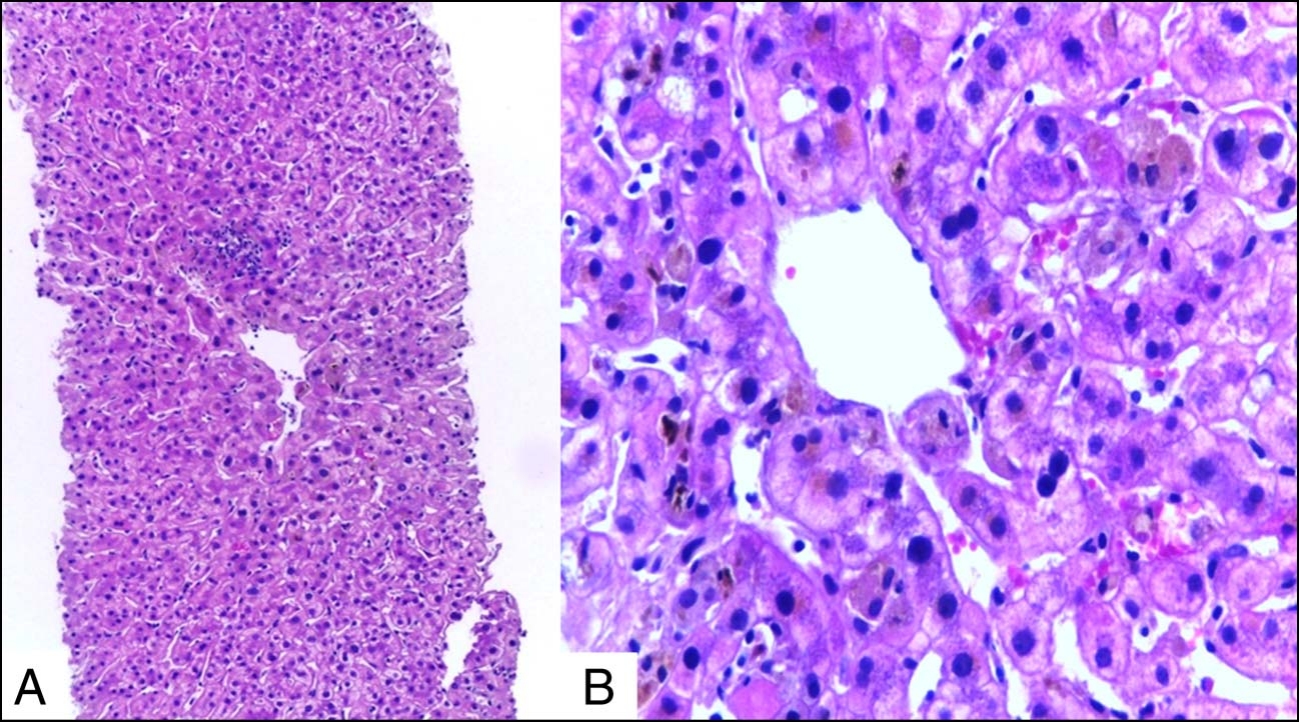
(A) Liver biopsy with mild centroacinar inflammation and bile pigment deposits. Haematoxilin-eosin stain (HE) 100×. (B) Liver biopsy showing cholestasis within the hepatocyte cytoplasm and intracanalicular bile thrombi. HE, 400×.

To evaluate the cause of cholestasis, a percutaneous liver biopsy was performed before surgery. Bland lobular cholestasis with a normal portal tract was observed without evidence of liver fibrosis (Figure 1).

**Figure 2.**
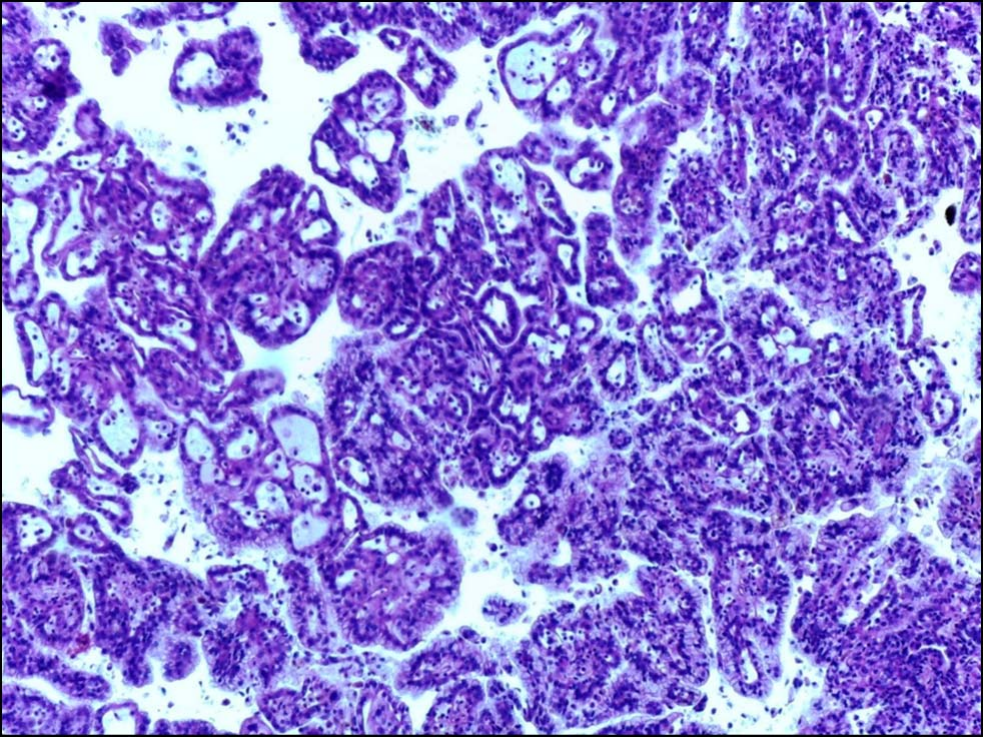
Type 1 papillary renal cell carcinoma. Haematoxilin-eosin stain, 100×.

Subsequently, right nephrectomy was performed and revealed a type 1 pRCC (Figure 2). After surgical tumor removal, a clear improvement of pruritus and jaundice was observed. A significant decrease in BA levels and liver biochemistry followed (7-day postoperative total bilirubin: 1.7 mg/dL, conjugated bilirubin: 1.6 mg/dL, aspartate aminotransferase: 64 UI/mL, alanine aminotransferase: 22 UI/mL, alkaline phosphatase: 199 UI/mL, gamma glutamyl transferase: 167 UI/mL, BA 1.4 μmol/L). He was discharged home; as an outpatient, he continued to have improvement of liver chemistries that persisted 30 days after surgery.

## DISCUSSION

Paraneoplastic intrahepatic cholestasis was first described by Dr. Stauffer, in 5 patients with renal cell carcinoma (RCC) and abnormal liver biochemistry with predominance of cholestasis, hepatosplenomegaly, and nonspecific hepatitis, in the absence of tumor infiltration and intrinsic liver disease.^3^ Another significant feature of the syndrome is the resolution of cholestasis after treatment of the neoplasm.^9–11^ The reappearance of cholestasis in conjunction with tumor relapse has been reported as well. After its description, SS has been associated with other neoplasms such as prostate adenocarcinoma, sarcoma, bronchial carcinoma, urothelial carcinoma, ovarian dysgerminoma, pancreatic adenocarcinoma, and Hodgkin lymphoma.^5,6,12,13,15–18^ In 1997, the first 2 cases of icteric SS were reported; since then, there is a current tendency to classify SS as (i) typical or anicteric and (ii) icteric variants. The icteric variant represents the less frequent form of SS, which would have a worse prognosis.^9,14,19^

The pathophysiology of SS is not fully clarified and would result from the following proposed mechanisms: (i) release of humoral procholestatic substances by tumor cells, (ii) release of humoral factors by tumor microenvironment, and (iii) immune-mediated cholestatic injury.^14,20^ Increased levels of interleukin (IL)-6 and/or IL-18 have been associated with SS and could act synergistically.^14,20^ The production of IL-6 by RCC in vivo has shown a high correlation with paraneoplastic manifestations, such as fever, weight loss, elevated C-reactive protein, and haptoglobin.^21–23^ A complete resolution of the hepatic biochemical alterations in SS has been observed using anti-IL-6 monoclonal antibodies.^21^ It has been reported that renal tubular cells are the most prominent source of IL-18 in the presence of inflammation and infection.^24^ pRCC is a rare subtype of renal cell carcinoma, arising from the renal tubular epithelium that shows a papillary growth pattern. It could be speculated that tubular cells of the pRCC or its tumoral microenvironment release IL-18. This increase in IL-18 inhibits the hepatocyte-canalicular membrane transporter multidrug resistance-associated protein 2 mediated by NF-κB and Yin Yang 1, and its inhibition will cause cholestasis and eventually jaundice.^25,26^

Another mechanism of production of paraneoplastic syndrome is the loss of tolerance to autoantigens expressed by the neoplasm with the consequent autoimmune cross-reaction.^27^ These immunological mechanisms, in addition to liver involvement, can produce other paraneoplastic clinical manifestations such as polyneuropathies, rheumatological diseases, endocrinological diseases, and myositis.^27^ Unlike SS, cholestasis caused by an immune-mediated mechanism could be associated by the vanishing bile duct syndrome.^28,29^ This paraneoplastic variant is rare; its diagnosis relies on liver biopsy and has a poor prognosis.^29^ Vanishing bile duct syndrome is associated mainly with Hodgkin lymphoma and exceptionally with other conditions such as uterine cancer.^30,31^

The most frequent finding in liver histology in SS is the presence of cholestasis defined as canalicular or bland cholestasis.^20^ This type of injury, also observed in other liver conditions, is characterized by the presence of hepatocyte cholestasis and dilatation of the bile canaliculi with the presence of bile thrombus, but without evidence of ductal injury and interface hepatitis or portal inflammation.^13,14^

In this case report, the patient presented with a type 1 pRCC of the right kidney associated with symptomatic cholestasis due to the presence of jaundice and pruritus. Liver biopsy before surgery showed canalicular cholestasis. Surgical removal of the renal tumor produced a dramatic resolution of the clinical picture with improvement of biochemical markers of liver injury and serum levels of bile acids.

Stauffer syndrome currently lacks diagnostic criteria. However, clinical diagnostic workup should include (i) underlying diagnosis of malignancy, (ii) cholestatic liver damage, (iii) absence of liver metastasis or obstructive jaundice, (iv) other causes of intrahepatic cholestasis ruled out by medical history and serology, (v) liver biopsy that may be necessary for accurate differential diagnosis, and (vi) reversion of cholestatic markers after successful tumor response by surgical removal or systemic therapy.

## DISCLOSURES

Author contributions: JA Sorda, EG Ballerga, A. Avagnina, and FJ Barreyro wrote and approved the article. JA Sorda and FJ Barreyro reviewed the literature and revised the article for intellectual content. JA Sorda, EG Ballerga, G. Rojas, DA Grecco, and J. Daruich participated in clinical and surgical treatment. A. Avagnina and A. Paes evaluated liver and renal biopsies. FJ Barreyro is the article guarantor.

Financial disclosure: None to report.

Informed consent was obtained for this case report.
